# Lowered levels of nicotinic acetylcholine receptors and elevated apoptosis in the hippocampus of brains from patients with type 2 diabetes mellitus and *db/db* mice

**DOI:** 10.18632/aging.103435

**Published:** 2020-07-23

**Authors:** Yi Xu, Kun Cao, Bing Guo, Jie Xiang, Yang-Ting Dong, Xiao-Lan Qi, Wen-Feng Yu, Yan Xiao, Zhi-Zhong Guan

**Affiliations:** 1 Departments of Pathology at Guizhou Medical University and the Affiliated Hospital of Guizhou Medical University, Guiyang 550004, P. R. of China; 2 Key Laboratory of Endemic and Ethnic Diseases, Guizhou Medical University of the Ministry of Education, Guiyang 550004, P. R. of China; 3 Department of Pathophysiology, Guizhou Medical University, Guiyang 550004, P. R. of China; 4 Provincial Key Laboratory of Medical Molecular Biology, Guiyang 550004, P. R. of China

**Keywords:** type 2 diabetes mellitus, postmortem human brain, mice *db/db*, nicotinic acetylcholine receptors, learning and memory

## Abstract

Cognitive impairment caused by diabetes has been gradually recognized. Generally, nicotinic acetylcholine receptors (nAChRs) play an important role in the pathogenesis in dementia disorders including Alzheimer's disease (AD). However, the expression of nAChRs in the brains of type 2 diabetes mellitus (T2DM) is unexplored. This study explored the alterations of nAChRs in the postmortem brains of patients with T2DM and brains of *db/db* mice. Morris water maze test was used to appraise the ability of spatial learning and memory; Western blotting and RT-qPCR were performed to determine the expressions of target protein and mRNA, respectively; TUNEL was used to detect the apoptosis of neurons. We found that the protein levels of nAChR α7 and α4 subunits were significantly decreased and the apoptosis rates in neurons elevated in the hippocampus of T2DM patients and *db/db* mice as comparison to controls. Furthermore, the *db/db* mice exhibited the impaired cognition, the elevated level of pro-apoptotic protein and the reduced level of anti-apoptotic and synaptic proteins. This study shows the lowered level of nAChR α7 and α4 subunits and the elevated apoptosis in the hippocampus of T2DM patients and *db/db* mice, which might help explain the impaired cognition in T2DM.

## INTRODUCTION

The incidence of diabetes mellitus, the most common metabolic disorder worldwide, is dramatically increasing. It has been estimated that 451 million people between the ages of 18-99 were afflicted in 2017 and a number that is expected to rise to 693 million by 2045 [1]. The two major subtypes of DM, type 1 (T1DM) and type 2 (T2DM), account for approximately 7-12% and 87-91% of all cases, respectively [2].

T2DM is characterized by insulin resistance and/or impaired insulin secretion, resulting in hyperglycemia. In recent decades, due to the improvement of blood glucose control and additional treatment strategies for diabetic complications, the treatment of typical diabetic complications (including cardiovascular disease, peripheral neuropathy, retinopathy and nephropathy) has been significantly improved, so the life span of diabetic patients has been greatly extended. However, other diabetes related factors downstream of chronic hyperglycemia may gradually affect the brain over time, including cognitive impairment, neurophysiological and structural changes in the brain. Indeed, T2DM is a risk factor for developing dementia, including Alzheimer’s disease (AD) [3–5]. The mechanisms by which cognitive abilities are impaired by diabetes have not yet been clearly established. However, altered neurogenesis, electrophysiological deficits, injury due to oxidative stress, neuroinflammation and neuronal apoptosis may be involved [6–8].

AD, the most common form of dementia, is characterized by loss of memory, confusion and impairment of cognitive function. Early neurobiochemical findings on AD revealed impaired cholinergic transmission due to the reduced activity of nicotinic acetylcholine receptors (nAChRs) [9, 10], as well as fewer numbers of these receptors in the cortex and hippocampus [11, 12]. Actually, nAChRs are members of the cysteine-loop family of ligand-gated ion channels, and to date, a total of 17 subunits (α1-α10, β1-β4, γ, δ, and ε) have been identified [13]. In the case of the mammalian brain, 12 different nAChR subunits have been detected (α2-α10 and β2-β4) [14]. These receptors comprised of five subunits arranged around a pore that functions as an ion channel. Neuronal nAChRs are either homomeric, containing with five α subunits, or heteromeric, with a combination of α and β subunits from two different subfamilies. The two major subtypes of nAChRs expressed in the mammalian central nervous system (CNS) are the heteromeric α4β2 nAChRs and homomeric α7 nAChRs [15, 16], both of which are expressed of high levels in the hippocampus, cortex, thalamus, ventral tegmentum and striatum [17].

In the CNS, nAChRs are expressed in the postsynaptic, presynaptic and axonal regions of neurons. Interestingly, extensive investigations indicated that nAChRs are important regulators of memory, learning, locomotion, attention and addiction [18–21]. Moreover, stimulation of nAChRs may protect against the toxic effects of β-amyloid peptide through activation of phosphatidylinositol 3-kinase/protein kinase B axis and the anti-apoptotic factor B-cell lymphoma-2 (Bcl-2), as well as down-regulation of glycogen synthase kinase-3 (GSK3) [22]. Over-activation of GSK3 is associated with high levels of toxic β-amyloid oligomers, hyperphosphorylated tau and neurofibrillary tangles [23, 24]. In addition, activation of nAChRs is also anti-inflammatory by down-regulating nuclear factor-kappa B via Janus kinase 2 [25]. Moreover, oral administration of the selective α7 nAChR agonist TC-7020 to *db/db* mice reduced their weight gain, food intake and blood levels of glucose, glycosyl hemoglobin (HbA1c), and triglyceride [26]. Recently, the beneficial effects of cholinergic stimulation in alleviating neuroinflammation and metabolic derangements associated with obesity have been demonstrated [27].

To date, the possibility concerning whether the expression and composition of nAChRs in pivotal regions of the brain (e.g., the cerebral cortex and hippocampus) is influenced by T2DM remains unexplored, although this might lead to novel pharmacological treatment of T2DM. The current investigation was designed to compare nAChR α4, α7 and β2 subunits in the postmortem brains of patients with T2DM and age-matched control individuals, as well as in an animal model of T2DM (*db/db* mouse) and non-diabetic control (*db*/*m* mouse).

## RESULTS

### Expression of nAChR subunits at protein level in postmortem brain tissues

**Figure 1:**
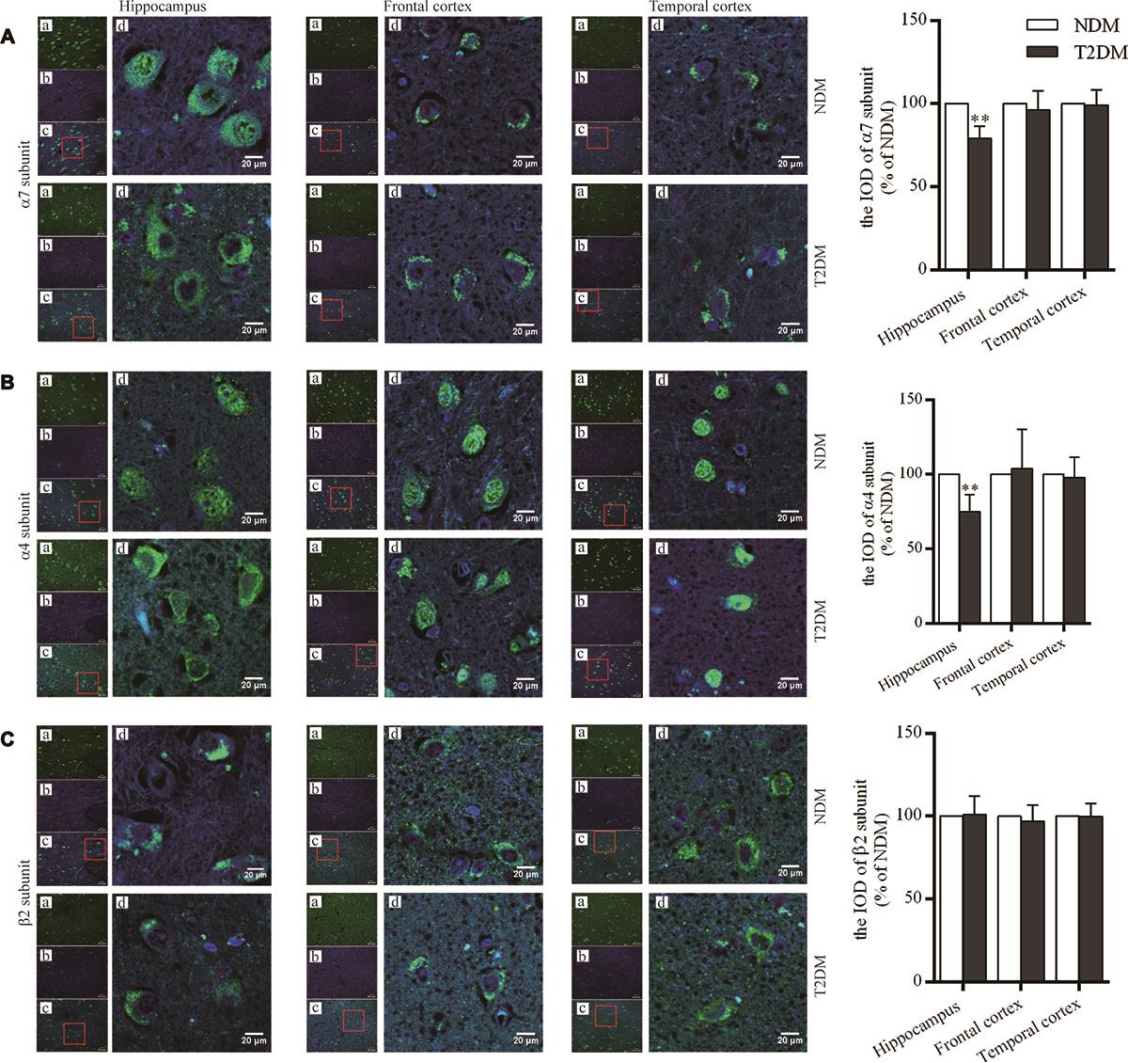
Immunofluorescent staining for nAChR α7 (**A**), α4 (**B**) and β2 (**C**) subunits in the hippocampus (CA3), and the frontal and temporal cortices of patients with T2DM (n=6) and age-matched controls (NDM, n=6). Photographs were taken by using a laser confocal microscope. The α7, α4 and β2-positive neurons were reacted by specific antibodies as shown as green (a); cell nuclei are stained as blue (using DAPI) (b); a and b were merged as one picture (c); and a partial area from c was selected to be magnified (d) with scale bars=20 μm. The values presented as percentage of the control by relative quantification for α7, α4 or β2 subunit staining in those regions are means ± SEM; **p<0.01 as compared to NDM employing the two-tailed unpaired Student’s *t* test.

The levels of nAChR α7 and α4 subunit proteins in the hippocampus (CA3, which is well-known as one of the areas as most critical to cognitive function) were significantly lower in the T2DM than control samples (α7 subunit: 100.0±5.1% in NDM, 79.2±2.9% in T2DM, F=1.522, *p*<0.01; α4 subunit: 100.0±4.7% in NDM, 74.8±4.7% in T2DM, F=0.008, *p*<0.01), but no significant differences in these levels in the frontal and temporal cortices were observed (Figure 1A, 1B). For the levels of nAChR β2 subunit protein, no significant changes were found in the hippocampus (CA3), and the frontal and temporal cortices in the T2DM as compared to control samples (Figure 1C).

### The Morris Water Maze (MWM) test to *db*/*db* mice and controls

**Figure 2:**
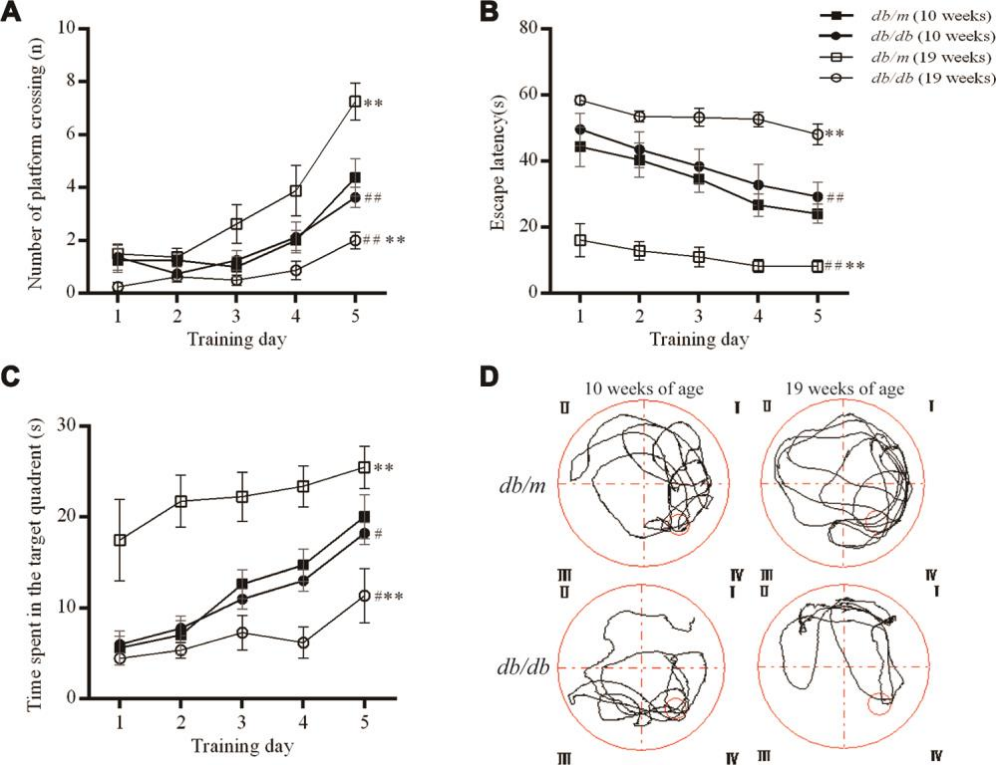
**Evaluation of the spatial learning and memory of *db/db* (n=8) and *db/m* (n=8) by MWM test.** (**A**) The number of platform crossings (n); (**B**) escape latency (s); (**C**) times spent in the target quadrant (s); (**D**) typical swimming paths to reach the original position of the platform. The values presented are mean ± SEM. **p<0.01, #*p*<0.05 and ## *p*<0.01 as determined by the two-tailed unpaired Student’s *t* test.

The MWM test reflects the spatial learning and memory ability of experimental animals. When the mice were ten weeks old, there were no significant differences in the number of platform crossings, the times spent in the target quadrant or the escape latency between the *db/db* mice and controls. However, at 19 weeks of age, the number of platform crossings (7.2±0.7 in *db/m* group, 2.0±0.3 in *db/db*, F=3.904, *P*<0.01; 3.6±0.4 in *db/db* at 10 weeks, 2.0±0.3 in *db/db* at 19 weeks, F=0.269, *P*<0.01) and the times spent in the target quadrant (25.5±2.3s in *db/m*, 10.7±2.6s in *db/db*, F=0.111, *P*<0.01; 18.2±1.2s *in db/db* at 10 weeks, 10.7±2.6s in *db/db* at 19 weeks, F=3.039, *P*<0.05) were significantly lower in *db/db* mice as compared to controls. The escape latency (8.2±1.8s in *db/m*, 48.1±3.1s in *db/db*, F=2.879, *P*<0.01; 26.3±4.3 *in db/db* at 10 weeks, 48.1±3.1s in *db/db* at 19 weeks, F=0.520, *P*<0.01) was significantly longer in the *db/db* group as compared to the *db*/*m* mice and also the corresponding *db/db* values at 10 weeks of age (Figure 2). Thus, the diabetic mice exhibited age-related impairment of spatial learning and memory.

### Expressions of nAChR subunits at protein and mRNA levels in the mouse brains

**Figure 3:**
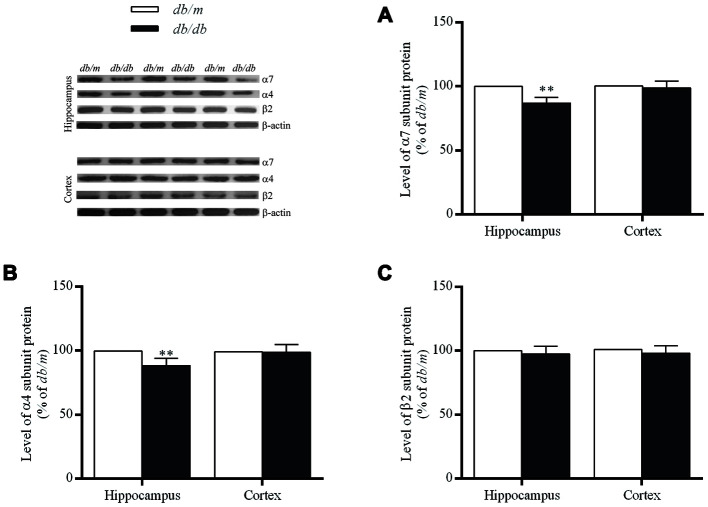
****The levels of nAChR α7 (**A**), α4 (**B**) and β2 (**C**) subunit proteins in the hippocampus and cortex of *db/db* and *db/m* mouse brains as determined by Western blotting. The values presented as percentage of the control by relative quantification are mean ± SEM (n=8 for each group). **p<0.01 as compared to *db/m* mice determined by the two-tailed unpaired Student’s *t* test. Representative Western blots are displayed in the upper left corner of the figure.

**Figure 4:**
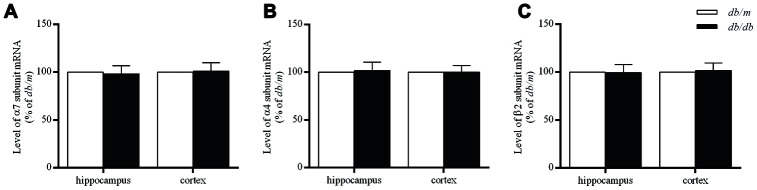
****The levels of mRNA encoding nAChR α7 (**A**), α4 (**B**) and β2 (**C**) subunits in the hippocampus and cortex of *db/db* and *db/m* mouse brains as determined by RT-qPCR. The values presented as percentage of the control by relative quantification are mean ± SEM (n=8 for each group). Application of the two-tailed unpaired Student’s *t* test revealed no significant differences.

The levels of α7 and α4 subunit proteins in the hippocampus of *db/db* mouse brains were significantly lower than those in *db*/*m* mice (α7 subunit: 100.0±1.7% in *db/m*, 86.9±1.6% in *db/db*, F=0.022, *p*<0.01; α4 subunit: 100.0±1.8% in *db/m*, 88.3±2.0% in *db/db*, F=0.158, *p*<0.01) (Figure 3A and 3B). Whereas, no significant differences of α7 and α4 proteins were determined in the case of the cortices (Figure 3A and 3B) between two groups or in the corresponding levels of mRNA (Figure 4). On the other hand, the levels of β2 subunit protein both in the hippocampus and cortices were no significant changes in *db/db* mice compared to *db*/*m* mice (Figures 3C and 4).

### Apoptosis in the brains of patients with T2DM and *db*/*db* mice

**Figure 5:**
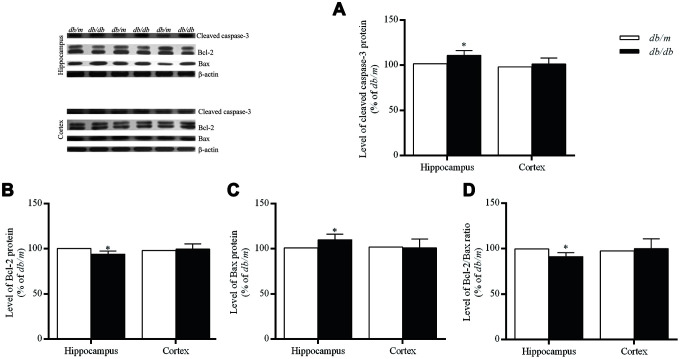
**Levels of apoptosis-related protein expressions in the hippocampus and cortex of *db/db* and *db/m* mouse brains as determined Western blotting.** (**A**) cleaved caspase-3; (**B**) Bcl-2; (**C**) Bax; (**D**) the Bcl-2/Bax ratio. The values presented as percentage of the control by relative quantification are mean ± SEM (n=8 for each group). *p<0.05 as compared to *db/m* mice as determined by the two-tailed unpaired Student’s *t* test. Representative Western blots are displayed in the upper left corner of the figure.

Apoptosis, a type of programmed cell death involving characteristic morphological and biochemical changes, is executed by a number of different proteins. The rate and extent of apoptosis are frequently characterized determined on the basis of the activity of caspases, proteases that cleave specific substrates. The Bcl-2 family concerning cell apoptosis includes anti-apoptotic Bcl-2 and pro-apoptotic Bcl-2 associated X protein (Bax) [28]. In the hippocampus of the *db/db* mice, cleaved caspase-3 and Bax were significantly increased as compared with controls (cleaved caspase-3: 100.0±3.5% in *db/m*, 110.9±1.9% in *db/db*, F=2.094, *p*<0.05; Bax: 100.0±2.7% in *db/m*, 109.9±2.3% in *db/db*, F=0.602, *p*<0.05) (Fig 5). Whereas, Bcl-2 (100.0±2.0% in *db/m*, 93.9±1.2% in *db/db*, F=0.905, *p*<0.05) and Bcl-2/Bax ratio (100.0±2.4% in *db/m*, 91.4±1.5% in *db/db*, F=4.282, *p*<0.05) in *db/db* group were significantly decreased as compared to controls (Figure 5). However, no such differences in case of the cortex were found between the two groups (Figure 5).

**Figure 6:**
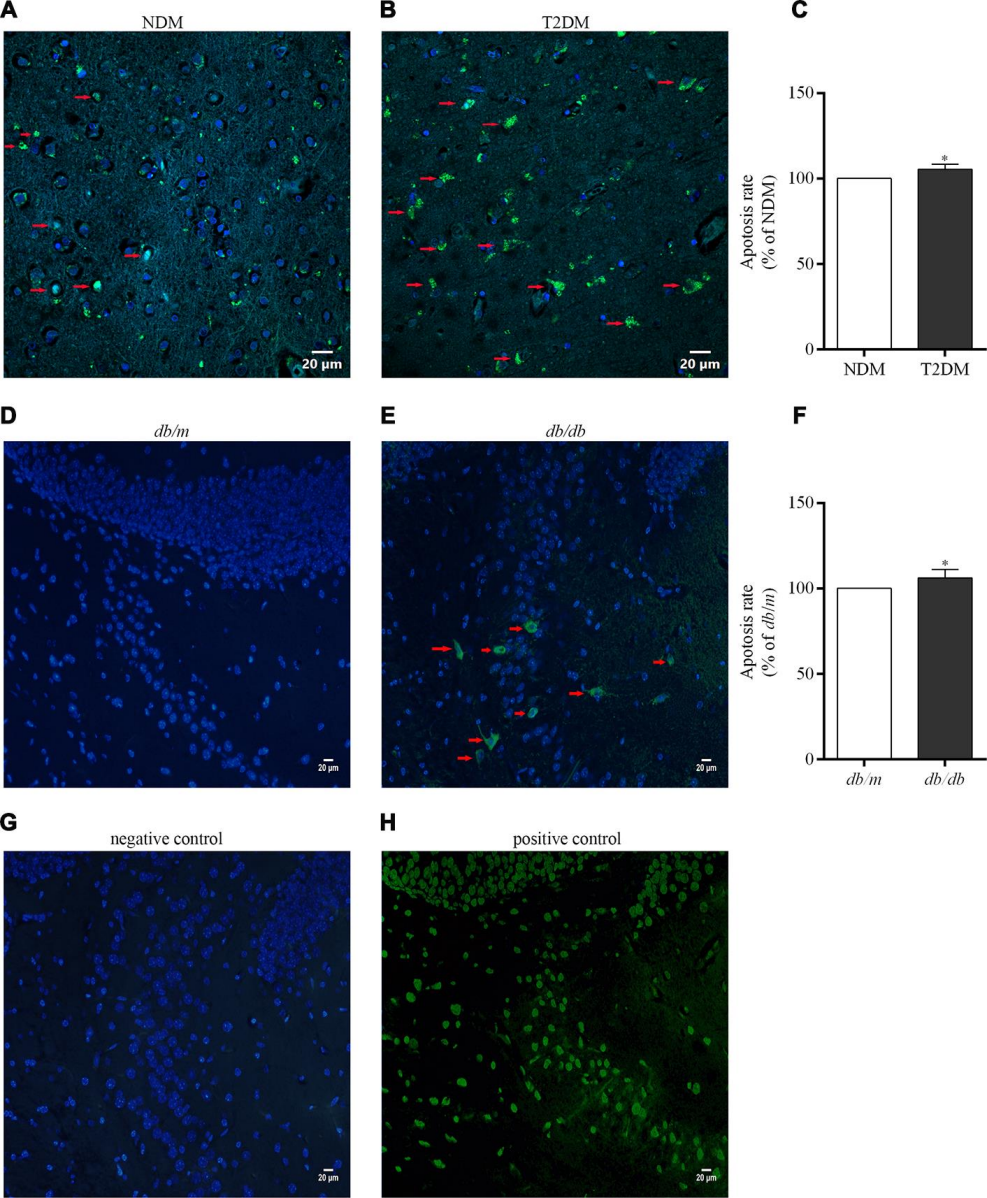
**Apoptotic cells in the brains of the patients with T2DM and *db/db* mice as well as controls *in situ* detected with the TUNEL assay.** Photographs were taken by using a laser confocal microscope (scale bars=20 μm). The co-localization of nuclei (DAPI, blue) and TUNEL-positive cells (green) indicated by red arrow are shown in the merged images. (**A**) NDM; (**B**) T2DM; (**C**) apoptosis rate of neurons in the human brains; (**D**) *db/m* mice; (**E**) *db/db* mice; (**F**) apoptosis rate of neurons in the mouse brains; (**G**) negative control for the method; (**H**) positive control for the method. The values presented as percentage of the control by relative quantification are mean ± SEM (n=5 for each group). *p<0.05 as compared to NDM (**C**) or *db/m* mice (**F**) as determined by the two-tailed unpaired Student’s *t* test.

Detection of DNA fragmentation by the terminal deoxynucleotidyl transferase-mediated nick end labeling (TUNEL) technique is a standard and reliable histochemical approach for detecting and quantitating cells in later stages of apoptosis [29]. Here, an elevated neurons apoptosis rate in the hippocampus of patients with T2DM and *db/db* mice was determined as compared to controls (100.0±1.3% in NDM, 105.3±2.0% in T2DM, F=0.000, *p*<0.05; 100.0±1.3% in *db/m*, 106.2±0.9% in *db/db*, F=2.192, *p*<0.05) (Figure 6).

### Levels of synaptic proteins in the brains of *db*/*db* and *db*/*m* mice

**Figure 7:**
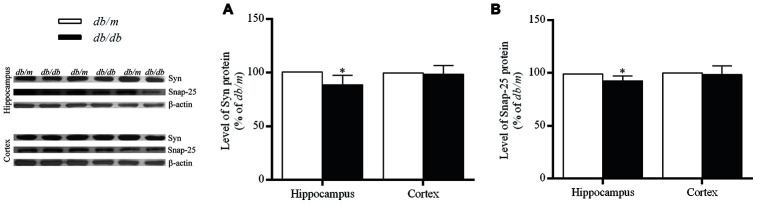
**The levels of synaptic proteins in the hippocampus and cortex of *db/db* and *db/m* mouse brains as determined by Western blotting.** (**A**) Syn; (**B**) Snap-25. The values presented as percentage of the control by relative quantification are mean ± SEM (n=8 for each group). *p<0.05 as compared to *db/m* mice as determined by the two-tailed unpaired Student’s *t* test. Representative Western blots are displayed in the left site of the figure.

The levels of synaptic proteins including synaptophysin (Syn) and 25-kD synaptosomal protein (Snap-25) were significantly lower in the hippocampus of *db/db* mice than those of *db*/*m* mice (Syn: 100.0±3.1% in *db/m*, 88.7±3.1% in *db/db*, F=0.003, *p*<0.05; Snap-25: 100.0±1.7% in *db/m*, 92.4±1.7% in *db/db*, F=0.034, *p*<0.05) (Figure 7), while no difference was found in the case of the cortex.

### The relationship between the levels of nAChR subunits and Bcl-2/Bax Syn and Snap-25 in the hippocampus of the brain of *db*/*db* mice

**Figure 8:**
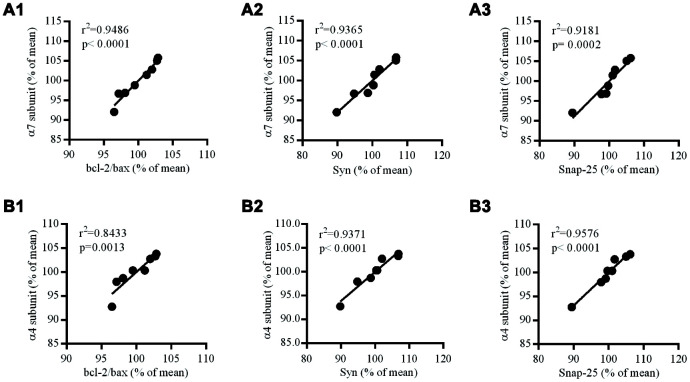
**Correlation between nAChR subunits and Bcl-2/Bax ratio, Syn or Snap-25 in the hippocampus of *db/db* mouse brains.** (**A1**–**3**) the correlation between α7 and Bcl-2/Bax, Syn or Snap-25; (**B1**–**3**) the correlation between α4 and Bcl-2/Bax, Syn or Snap-25. The values presented are mean ± SEM (n=8) as determined with the Pearson correlation test.

In the hippocampus of the brains of *db/db* mice, the levels of nAChR α7 and α4 subunits at protein levels were positively correlated to the levels of Bcl-2/Bax, Syn and Snap-25 (Figure 8).

## DISCUSSION

In the present study, the levels of nAChR α7 and α4 subunit proteins (but no β2) in the hippocampus (CA3) of patients with T2DM were significantly lower than those in age-matched controls, with no significant differences in the case of the frontal or temporal cortex, indicating region specific alterations and subunit changes of the receptors caused by this disorder. In our earlier reports in AD patients as compared with age-matched controls, the level of α4 subunit protein was reduced significantly both in the hippocampus and temporal cortex; the level of α7 was obviously decreased in the hippocampus but no significant change in the temporal cortex; and the level of β2 in either the hippocampus or the temporal cortex was no significant difference [11]. Thus, the alterations associated with AD and T2DM differed somewhat; furthermore, in both cases the levels of the α7 and α4 subunits in the hippocampus, one of the most important tissues of the brain limbic system and critical for learning and memory, were affected.

In the current investigation, we found that the spatial learning and memory (as determined with the MWM test) of *db/db* mice did not differ significantly from that of *db*/*m* mice at 10 weeks of age, but were gradually impaired with the prolongation of diabetes, in agreement with a previous report [30]. These results further confirm that a deficiency in the leptin receptor may not be the primary cause of cognitive decline in *db/db* mice and that T2DM leads to gradual cognitive impairment in these animals. Previous studies have also found that the novel object recognition ability of *db/db* mice is declined [31]. However, the *db/db* mice did not show apparent working memory disturbance in the spatial working memory version of the MWM or in the radial water maze [32], which may need a further investigation.

In the hippocampus of *db/db* mice with cognitive impairment, the levels of α7 and α4 subunits were significantly lower than those in *db*/*m* mice, with no significant difference between those groups in the case of the cortex; and the level of β2 subunit in the hippocampus and cortex of *db/db* mice was no significant difference as compared with *db*/*m* mice. These changes were thus consistent with those observed in the postmortem brains of patients with T2DM.

At the same time, there were no differences in the levels of mRNAs encoding α7, α4 and β2 subunits in the hippocampus and cortex of the two groups of mice, indicating that the deficit of nAChRs in the *db/db* mouse brain may be related to changes in synthesis, posttranslational modifications, and/or turnover (including membrane insertion). Hellstrom-Lindahl and colleagues [33] compared the regional expression of mRNA encoding the α4 and α7 subunits in postmortem brain tissues from patients with AD and controls and observed no differences in the case of α4 mRNA in any of the regions analyzed, whereas the level of α7 mRNA was significantly higher only in the hippocampus of AD brains. Here, our results indicate that the decrease in the number of nAChRs reflects a post-transcriptional event.

In the hippocampus of *db/db* mice, cleaved caspase-3 and Bax were up-regulated, while Bcl-2 down-regulated and the Bcl-2/Bax ratio reduced in comparison to *db*/*m* mice; and the number of TUNEL-positive cells elevated both in the hippocampus of patients with T2DM and *db/db* mice comparison to controls, indicating enhanced apoptosis by the disorder, similar to previous report [34].

Syn is closely related to the regulation of synaptic structural and functional plasticity, in which numerous proteins in presynaptic vesicles play important roles in the circulation of these vesicle and release of neurotransmitters. Since the levels of Syn can reflect the density and number of synapses, this level is often used as an indicator of synaptic integrity. Snap-25 mediates primarily the anchoring and fusion of synaptic vesicles and thereby plays a key role in the release neurotransmitters. In the present study, the levels of both Syn and Snap-25 in the hippocampus of *db/db* mice were significantly decreased, indicating a reduction in the number of synapses. Moreover, the levels of the α7 and α4 subunit proteins were significantly positively correlated to those of Bcl-2/Bax, Syn, and Snap-25, individually. Loss of neurons and a reduction in the number of synapses are also seen in many areas of the cortices of patients with AD [35, 36]. In addition, Ramos-Rodriguez and co-workers [37] reported that pre-diabetes and T2DM both promoted neuronal cell apoptosis and synaptic loss in a mouse model of AD. Accordingly, these phenomena might give an explanation to the reductions in the levels of nAChR subunits.

Accumulating evidence indicates that nAChRs are essential for optimal performance of numerous cognitive processes [18–21]. For instance, nicotine and inhibitors of cholinesterase improve cognitive function in patients suffering from neurodegenerative disorders such as AD [21, 38]. Moreover, as recently demonstrated, galantamine (a cholinesterase inhibitor) also exerts beneficial anti-inflammatory and metabolic effects in patients with metabolic syndrome [39]. Such findings indicate that region-specific alterations in the levels of α7 and α4 subunits in the brain might account for some of the functional disorders associated with T2DM and that nAChRs are probably an attractive therapeutic target for treatment of this disease. In summary, we document here region-specific alterations in the levels of nAChR α7 and α4 subunit proteins, both in the brains of patients with T2DM and *db/db* mice, which might help explain the impaired cognition in T2DM.

## MATERIALS AND METHODS

### Samples of human brains

Postmortem human brain samples were provided by the Netherlands Brain Bank (Amsterdam, the Netherlands). Material Transfer Agreement and Implementing Letter Regarding Project (No.1060) were granted by the Netherlands Brain Bank and ethical permission for the study by the Ethical Committee of Guizhou Medical University (No. 2018-067). The hippocampus and frontal and temporal cortices were from six patients with a clinical diagnosis of T2DM, and from six subjects (no-diabetes mellitus, NDM) with no known history or symptoms of this disease. The case histories of these subjects in the study are listed in Table 1.

**Table 1:** Characteristics of the patients with T2DM and control individuals (NDM).

	**T2DM**	**NDM**
Cases	6	6
Age at times of death (years, mean±SD)	78.67±1.51	77.33±1.97
Sex (M/F)	3/3	3/3
Postmortem delay (h)	6.1±0.9	7.2±1.6
Tissue pH	6.48±0.3	6.65±0.36
Brain weight (g)	1191.67±147.71	1280.67±116.49
Duration of T2DM (years)	9±2.7	not applicable

Notice: T2DM, type 2 diabetes mellitus; NDM, no-diabetes mellitus.

### Experimental animals

Male eight-week-old C57BLKS/J-*db/db* mice (n=8) and their age-matched non-diabetic *db*/m littermates (n=8) were purchased from the Model Animal Research Center of Nanjing University, China (License No. SCXK (Su) 2018-0008, Certificate No. 201804455). All were housed at 22-25°C with a 12 h light/dark cycle and access to food (a normal chow diet (NCD) with 5% fat (8.5 KJ/g) and water in the SPF (specific pathogen-free) Animal Laboratory Center of Guizhou Medical University. The experiments described here were pre-approved by the Ethical Committee of Guizhou Medical University, China (No. 1800456).

In preclinical diabetic research, *db/db* mice are commonly used as a model [40]. It should be noted, however, that *db/db* mice are deficient in leptin receptors and leptins is involved in the development of the brain of mouse embryos [41]. Nonetheless, this deficiency in leptin receptors appears not to be the main cause of cognitive decline in these animals [42, 43]

### The Morris water maze (MWM) test

When the mice were 10 weeks old, the MWM test was conducted to evaluate their spatial learning and memory as reported previously [44]. In brief, this test was performed in a circular pool with a diameter of 110 cm and a height of 30 cm, filled with opaque water at 26°C. An escape platform with a diameter of 7 cm was submerged 1 cm below the surface of the water. During four consecutive days of training (four trials per day), mice which could not find this escape platform within 60 s were guided to it. Thereafter, the mice were placed at the same starting location and subjected to a 60 s trial without the escape platform. Their behavior was tracked and recorded by an overhead video camera and a computer system equipped with ‘Viewer 2’ software (Biobserve GmbH, Bonn, Germany) in order to calculate the time required to swim to the original position of the platform, as well as the number of passes over and time spent at this position. Eight weeks later, the MWM test was conducted again.

### Immunofluorescent staining

Immunofluorescent staining and quantitation of α4, α7 and β2 subunits in the brains of samples were performed as described previously [45]. In brief, sections were first heated at 58°C for 1 h and thereafter deparaffinized and hydrated. After three washes in phosphates buffered saline (PBS) and microwaving in 0.01 M citric acid buffer (pH 6.0) for 20 min to achieve antigen repair, the sections were treated with normal goat serum for 1 h and then incubated with antibodies against α4 (1:250), α7 (1:250), or β2 (1:200) (all obtained from Gene Tex, USA) overnight at 4°C. The following day, these samples were washed three times with PBS and incubated with secondary antibodies labeled with Alexa Fluor 488 (FITC, green, Thermo Fisher Scientific) for 1 h at room temperature, followed by washing with PBS and staining of nucleus with DAPI (blue, Vector Laboratories, Inc. USA) for 5 min. Digital images were collected with an epifluorescence microscope (Nikon, Japan) were captured using Nis-Elements D software (Nikon, Japan). For each sample, ten cells chosen at random were counted. The threshold for integrated optical density was defined automatically by Image-Pro Plus 6.0 software, which compares the optical density of all samples to the same standard.

### Preparation of samples of mouse brains

At nineteen weeks age, mice were euthanized with a lethal injection of sodium pentobarbital (200 mg/kg BW) and their brains then dissected out immediately. Each brain was first divided into its left and right hemispheres along the sagittal median line, after which the hippocampus and cortex were dissected out of the left hemisphere. Portions of these tissues were placed into RNAlater (Ambion, Thermo Fisher Scientific) for analysis of gene expression by Real-time qPCR, while other portions were snap-frozen in liquid N2 and stored for subsequent Western blotting. The right hemisphere was fixed in 4% paraformaldehyde for routine histological and immunofluorescent examination.

### Western blotting

Samples of the left hippocampus or cortex were homogenized in PBS containing complete protease inhibitors in a glass vesicle; the resulting homogenate was centrifuged at 12,000 rpm at 4°C for 20 min; and the protein concentrations of the supernatant thus obtained determined with the BCA protein assay kit. Proteins (30 μg) were subsequently separated by 8-10% SDS-PAGE and then blotted onto polyvinylidene difluoride (PVDF) membranes with a transfer unit (Bio-Rad Inc.). For the relative quantification of the proteins, these membranes were thereafter incubated with antibodies against α7 (1:1000), α4 (1:1500) and β2 (1:1000), cleaved caspase-3 (1:1000, Gene Tex, USA), Bcl-2 (1:1000, CST, USA), Bax (1:1000, CST, USA), Snap-25 (1:1000, Gene Tex, USA), Syn (1:1000, Gene Tex, USA) or β-actin antibody (1:5000, Gene Tex, USA) at 4°C overnight. After washing, the membranes were incubated with horseradish peroxidase-conjugated secondary antibody (1:5000, Gene Tex, USA) for 60 min. Finally, these membranes were incubated in ECL Plus (Pierce, Thermo Fisher Scientific) reagent and the signals thus obtained visualized by exposure to hyper-performance chemiluminescence film for 30 sec to 5 min.

### Real-time quantitative PCR

The levels of mRNA encoding α7, α4 and β2 investigated using Real-time quantitative PCR were performed as described previously [46]. In brief, total RNA was extracted from the hippocampus and cortex with TRIzol reagent (Invitrogen, Carlsbad, USA) using a homogenizer, according to the manufacturer’s instructions. Reverse transcription was performed using Prime Script RT reagent kit (TAKARA BIO INC.). The real-time quantitative PCR was performed in the Step One Plus Real-Time PCR System (Life Technologies) with the ABI SYBR Green Master Mix (Applied Bio systems, Thermo Fisher Scientific). Eight mice from each group were tested in this manner and all reactions were analyzed in triplicate. Relative quantitation values were calculated using the 2-ΔΔCT method. The primer sequences utilized are shown in Table 2.

**Table 2:** Sequences of the primers employed.

**Gene**	**Primer Sequence (5′-3′)**	**NCBI Reference Number**
nAChR α4	F:CCGGAATTCCTCGTCTAGAGCCCGTTCTG	NM_01730.5
	R:CCGAAGCTTGTCCGCGTTGTTGTAGAGGA	
nAChR α7	F:CCGGAATTCTCATTCTTCTGAATTGGTGTGC	NM_007390.3
	R:CCGAAGCTTTCTCGTCCTCCAGATTCTCTTC	
nAChR β2	F:CCGGAATTCGGTGTTCCTGCTGCTCATCTC	NM_009602.4
	R:CCGAAGCTTCTCACACTCTGGTCATCATCT	
β-actin	F:CGTTGACATCCGTAAAGACC	NM_007393.5
	R:CTAGGAGCCAGAGCAGTAATC	

### Assessment of apoptosis

*In situ* detection of cells with DNA strand breaks in brain sections (fixed in paraformaldehyde and embedded in paraffin) was achieved by the terminal deoxynucleotidyl transferase (TdT)–mediated deoxy-UTP nick end labeling (TUNEL) technique [47] utilizing a TUNEL Apoptosis Detection kit (Alexa Fluor 488, Yeasen Biotech Co., Ltd. Shanghai, China). For the negative and positive controls carried out for all assays, tissue sections were processed in an identical manner, except that the TdT enzyme was replaced by the same volume of distilled water or DNA enzyme I, respectively. TUNEL-positive nucleus appeared green and staining with DAPI revealed all nucleus. Images were captured with a fluorescent microscope (Nikon, Japan).

### Statistical analyses

Statistical analyses were performed using GraphPad Prism 6.0 and SPSS 22.0 software (SPSS Inc., USA) and the values presented are mean±SEM. The two-tailed unpaired Student’s *t*-test was to applied compare group differences. Potential correlations between the levels of neurons expressing the nAChR α7 and α4 subunits, and Bcl-2/Bax, Syn and Snap-25 were examined with the Pearson correlation test. Differences of *p***<**0.05 was considered statistically significant.

### Ethics approval

Material Transfer Agreement and Implementing Letter Regarding Project (No.1060) were granted by the Netherlands Brain Bank and ethical permission for the study by the Ethical Committee of Guizhou Medical University (No. 2018-067); animal use for this study was approved by the Ethical Committee of Guizhou Medical University, China (No. 1800456).
